# Dynamic Calibration and Validation of an Accelerometer Force Balance for Hypersonic Lifting Models

**DOI:** 10.1155/2014/813759

**Published:** 2014-01-20

**Authors:** Prakash Singh, Sharad Trivedi, Viren Menezes, Hamid Hosseini

**Affiliations:** ^1^Department of Aerospace Engineering, Indian Institute of Technology Bombay, Powai, Mumbai 400076, India; ^2^Institute of Pulsed Power Science and Graduate School of Science and Technology, Kumamoto University, Kumamoto 860-8555, Japan

## Abstract

An accelerometer-based force balance was designed and developed for the measurement of drag, lift, and rolling moment on a blunt-nosed, flapped delta wing in a short-duration hypersonic shock tunnel. Calibration and validation of the balance were carried out by a convolution technique using hammer pulse test and surface pressure measurements. In the hammer pulse test, a known impulse was applied to the model in the appropriate direction using an impulse hammer, and the corresponding output of the balance (acceleration) was recorded. Fast Fourier Transform (FFT) was operated on the output of the balance to generate a system response function, relating the signal output to the corresponding load input. Impulse response functions for three components of the balance, namely, axial, normal, and angular, were obtained for a range of input load. The angular system response function was corresponding to rolling of the model. The impulse response functions thus obtained, through dynamic calibration, were operated on the output (signals) of the balance under hypersonic aerodynamic loading conditions in the tunnel to get the time history of the unknown aerodynamic forces and moments acting on the model. Surface pressure measurements were carried out on the model using high frequency pressure transducers, and forces and moments were deduced thereon. Tests were carried out at model angles of incidence of 0, 5, 10, and 15 degrees. A good agreement was observed among the results of different experimental methods. The balance developed is a comprehensive force/moment measurement device that can be used on complex, lifting, aerodynamic geometries in ground-based hypersonic test facilities.

## 1. Introduction 

A measurement technique developed for hypersonic ground-based test facilities requires a meticulous calibration and validation of its performance, as the technique operates on models with complicated flow fields within the stringent constraints of ultrashort duration. The constraints include impulse loading and vibration of the test facility and model mounts during the run. The impulse loading lasts for about a millisecond and the vibrations generated during the run do not subside within the period of impulse. The vibrations may influence the signal output if their frequency lies within the operating frequency bandwidth of the sensors. But, the frequency of vibrations in a metallic diaphragm-explosion-driven test facility, such as a shock tunnel, is generally very high and a measurement technique deployed can work successfully if the sensors have a moderate operating frequency. In other words, a mismatch must exist between the natural frequency of the model and the operating frequency of the sensors to carry out measurements in hypersonic impulse facilities [1].

Force balances are popular at measurement of forces and moments in hypersonic impulse facilities as the output of these is independent of spatial resolution, which reduces uncertainty in measurement. The balances work effectively on small, rigid models that are generally used in hypersonic test facilities. A small, rigid model has low mass and high natural frequency and thereby undergoes adequate displacement under the action of low dynamic pressure freestream and vibrates at a very high frequency during the run. Instrumenting such a model with sensors of moderately high operating frequency can work well in short-duration shock tunnels.

A force balance works by virtue of motion of the test model during the operation, and, hence, there has to be an arrangement to unrestrain the model during the test. The unrestrained model-balance assembly has to move as a single rigid body, which has to be ensured during calibration of the balance. The most effective method of force balance calibration is to load the model with a known force, obtain the corresponding output, generate a system response function relating the output to the input through a convolution technique, and use it as a combined sensitivity of the model-balance assembly to the applied load. The method was adopted by several researchers in the past [2–4] and was found effective on models for shock tunnel applications. Mee [2] and Abdel-jawad et al. [4] used the convolution technique to calibrate stress wave force balances instrumented with strain gauges, while Kulkarni et al. [3] used the technique on a single component accelerometer force balance to measure drag on a model in a shock tunnel. In a well-integrated model-balance assembly, the sensitivity of an accelerometer balance is in fact the sensitivity of the accelerometers embedded in the balance. The calibration in such a case just ensures a good layout of the assembly.

We developed an accelerometer-based balance to measure forces and moments on a lifting model for use in shock tunnels [5]. The test assembly consisted of a light model supported on a rigid sting through flexible rubber bushes. The bushes allowed the model to move freely under the action of aerodynamic forces during the flow. Several uniaxial accelerometers were embedded in the desired directions in the test assembly to generate system output corresponding to aerodynamic forces and moments on the model. Forces and moments were derived from the acquired system output using the system response functions and a deconvolution technique. The balance was tested for measurement of drag, lift, and rolling moment on a flapped, delta wing at a hypersonic Mach number of 8 in IIT Bombay-Shock Tunnel (IITB-ST). The above forces and moment on the model were also deduced from surface pressure measurements, under the same aerodynamic test conditions, using high frequency pressure transducers. The pressure measurements serve as a validation of the performance of the force balance under the given test conditions.

This paper presents the calibration and validation of the accelerometer force balance developed in-house for measurements in shock tunnels. The experimental data reduced using the balance theory and the analytical data deduced using the Newtonian theory for the test model, both reported in [5], are also included with the current results for comparison. The paper covers the performance of three components of the balance, namely, drag, lift, and the rolling moment.

## 2. Materials and Methods 

### 2.1. Test Facility

The hypersonic shock tunnel, IITB-ST, was driven by a 50 mm diameter (inner) shock tube that operated in a reflected mode. An aluminum diaphragm of 1.2 mm thickness was ruptured by pressurization of the driver gas in the shock tube to start it. A converging-diverging nozzle was attached to the end of the shock tube to expand the shocked test gas (air) to a free stream of Mach 8 in a test section of dimensions 300 × 300 × 450 mm. The free stream conditions for the present set of experiments are listed in Table 1. The shock tube reservoir, behind the reflected shock wave, was steady with a pressure of 5.1 bar for a duration of about 1.5–2 ms, which was the test time for the model. The average dynamic pressure for the tests was 2267 Pa, and the test duration provided approximately 20 flow-body passes for the model, which was sufficient for the establishment of complex flow phenomena.

### 2.2. Force Balance and Test Model

**Figure 1:**
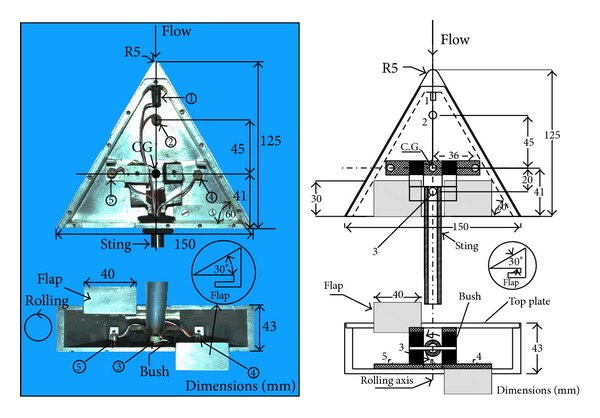
Photograph and schematic of test model assembly. Labels 1–5 indicate accelerometers.

The balance had four cube-shaped, soft rubber bushes of equal consistency that acted as a flexible suspension for the model during the hypersonic flow. The suspension allowed the model to be unrestrained in any direction during short-duration tests. The soft suspension isolated the model and the accelerometers from the mounting sting of the test assembly. Five uniaxial accelerometers of PCB Piezotronics (USA) were used in the balance to sense drag, lift, and rolling moment on the model, which made the assembly appropriate for this study. The properties of the accelerometers are given in Table 2. The test model was a blunt-nosed, delta wing with two rectangular flaps at its trailing end. The model was made of aluminum and weighed 0.517 Kg (floating mass on the suspension). The test model with the arrangement of the accelerometers is shown in Figure 1. The model chosen was light and rigid and had a natural frequency that was much higher than the loading frequency of the hypersonic flow. The model had a negative static margin longitudinally and rolled by virtue of the flaps at its trailing end. The location of the center of gravity (CG) of the model is indicated in Figure 1.

### 2.3. Dynamic Calibration

**Figure 2:**
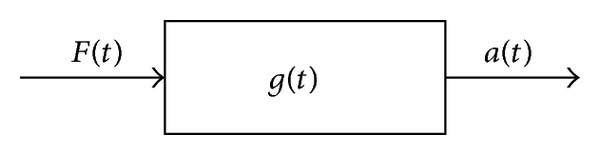
Schematic of linear, time-invariant system representing test model-accelerometer balance assembly.

The dynamic calibration of the balance involved charging the model-balance-sensor assembly (the system) with a known force/load using an impulse hammer and recording the time history of the response of the system to the applied impulse load. The system was assumed to be a linear, time-invariant dynamic system as shown in Figure 2, under the conditions of short test durations (1-2 ms) in the shock tunnel. If the applied impulse load is *F*(*t*) (Newton) and the corresponding output of the accelerometer in the balance is *a*(*t*) (volt), then the input and the output can be related by a convolution integral expressed as

(1) $$\begin{array}{c} a\left(t\right)=\int_{0}^{t}g\left(t-\tau \right)F\left(\tau \right)d\tau , \end{array}$$

where *g*(*t*) is called the impulse response function or the transfer function of the system that relates the input to the output.

During the calibration, the model/system was loaded with impulses of known magnitude that it was likely to encounter during the hypersonic flow in the tunnel. The impulse hammer with a metallic tip (PCB Piezotronics make; model no. 086C02) used for the application was equipped with a force sensor that transmitted the load-time history of the applied impulse. The system was fixed to its usual mount through the support sting, with the axis of the model aligned horizontally. For the axial force measurement, a uniaxial accelerometer was mounted in the system (accelerometer 1 in Figure 1) with its axis coinciding with the longitudinal axis of the test model. In order to simulate the axial force, the impulse load was applied at the nose of the model and was directed along its longitudinal axis. Two uniaxial accelerometers (2 and 3 in Figure 1) were mounted vertically on the bottom plate of the model, on either side of its CG along the longitudinal axis to sense normal force on the system. The impulse in this case was applied at the model CG, in the direction perpendicular to the longitudinal axis of the model. The test model was equipped with two rectangular flaps at the trailing end to generate roll, which was picked up by the vertical accelerometers 4 and 5, located along the transverse axis of the model. The flaps were the only source of roll on the system. The impulse load was applied at the location of accelerometer 4 on the lower flap, normal to the surface to simulate rolling moment during calibration. The accelerometers 4 and 5 could also sense the normal force applied at the CG. The applied impulse load covered the range of the actual aerodynamic load on the model during the entire operation, including at different model angles of attack (AOA) with the freestream. The estimates of the aerodynamic forces were obtained from the Newtonian theory [6].

The output of the impulse hammer and the system, which were force-time and acceleration-time histories, were recorded on the data acquisition system. The data was acquired at a frequency of 1 MHz. The operating frequency bandwidth of the accelerometers in the system was 1 Hz–10 kHz; hence the acquired signals were filtered at 10 kHz using a low pass filter for the analysis. The output of the hammer (input to the system) was *F*(*t*) and the output of the system was *a*(*t*), to be consistent with Figure 2. Fast Fourier Transforms (FFTs) were carried out on *F*(*t*) and *a*(*t*) in order to obtain *F*(*f*) and *a*(*f*), which are the transformed functions in frequency domain. The output of the system in frequency domain is the product of the system's impulse response and the transformed input. In other words, convolution in the time domain is equivalent to multiplication in the frequency domain. Therefore, the system response function can be expressed as

(2) $$\begin{array}{c} g\left(f\right)=\frac{a\left(f\right)}{F\left(f\right)}, \end{array}$$

where *g*(*f*) is the impulse response function of the system in frequency domain. A linear, time-invariant system always produces the frequency components that are present in its input. Hence, *F*(*f*) and *a*(*f*) are expected to have a common *x*-axis, which is the frequency. Having known *g*(*f*) and on obtaining *a*(*f*), the unknown transformed input to the system *F*(*f*) can be obtained in an actual aerodynamic operation through (2). An Inverse Fast Fourier Transform (IFFT) on *F*(*f*) can yield the unknown input *F*(*t*), which is the deconvoluted force-time history.

The aerodynamic forces reduced using the above method were with respect to the coordinates of the test model. The forces were resolved along and perpendicular to the freestream, based on the model AOA, to obtain drag (*D*(*t*)) and lift (*L*(*t*)) on the model.

### 2.4. Pressure Measurement

High frequency pressure sensors (MEAS France, model: EPIH-373-1.5B-/V5/L3 M/M) were used to acquire pressure-time history at various locations on the model surface. The model was rigidly fastened (without any soft suspension) through the sting in the tunnel test section during the pressure measurements. The diameter and length of the pressure sensors were 2 mm and 12 mm, respectively; the operating pressure limit (peak) was 1.5 bar while their sensitivities ranged from 14.5 to 17 mV/kPa. A total of 82 pressure taps were provided on the model with a good spatial resolution, covering all the likely complex flow pockets. The pressure transducers were piezoresistive, passive sensors, which were energized through a Dewetron (DEWE 31-32) power supply-cum-signal conditioner-cum-amplifier module. The available amplification factor on DEWE 31-32 varied from 0.25 to 40.

The measured surface pressure was resolved in the appropriate directions and was multiplied by the area of the domain of its influence to obtain the local aerodynamic forces. Summation of the local forces along the free stream and normal to the free stream gave the drag and the lift on the model, respectively. The rectangular flaps located at the trailing edge of the model were the only source of rolling. The net rolling moment on the model was obtained by the summation of the moments of all the contributing local pressure forces about the longitudinal axis of the model. Equations (3)–(5) are the expressions for drag, lift, and rolling moment on the model in terms of measured pressure. The force and moment coefficients are expressed by (6)–(8):

(3) $$\begin{array}{c} D\left(t\right)=\sum_{i=1}^{n}P_{i}A_{i}\sin\theta_{i}, \end{array}$$

(4) $$\begin{array}{c} L\left(t\right)=\sum_{i=1}^{n}P_{i}A_{i}\cos\theta_{i}, \end{array}$$

(5) $$\begin{array}{c} R\left(t\right)=\sum_{i=1}^{n}P_{i}A_{i}l_{i}\cos\theta_{i}, \end{array}$$

(6) CD=D(t)q∞Aref,

(7) CL=L(t)q∞Aref,

(8) CR=R(t)q∞ArefLref,

where *D*(*t*), *L*(*t*), and *R*(*t*) are the drag, lift, and rolling moment, respectively; *P*_*i*_ is the measured local pressure at any point *i* on the model surface; *A*_*i*_ is the area of action of *P*_*i*_; *θ*_*i*_ is the effective angle of resolution at any point *i* with respect to the freestream; *l*_*i*_ is the arm for the local moment at any point *i*; *C*_*D*_, *C*_*L*_, and *C*_*R*_ are the coefficients of drag, lift, and rolling moment, respectively; *q*_*∞*_ is the freestream dynamic pressure; and *A*_ref_ and *L*_ref_ are the model base area and base length, respectively.

## 3. Results and Discussion

**Figure 3:**
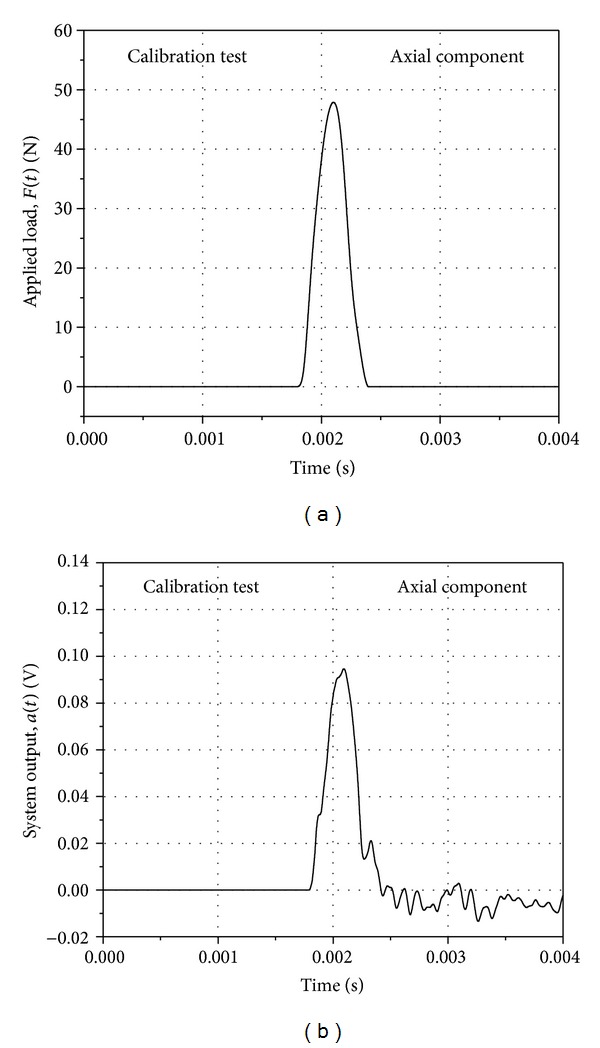
Calibration signals, (a) applied load and (b) system output signals for axial component.

**Figure 4:**
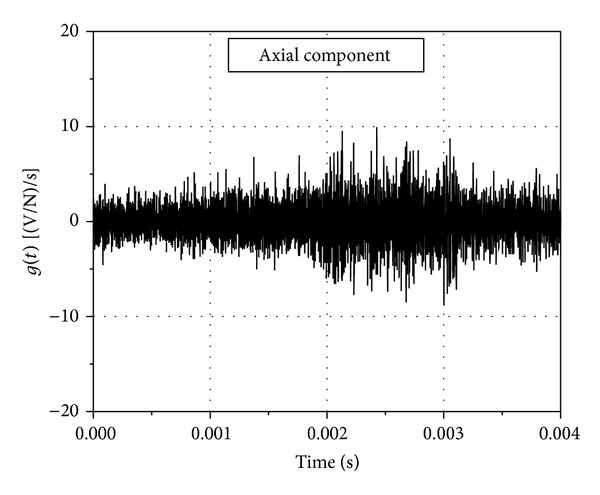
System response function for axial component.

The representative signals obtained during the generation of the impulse response function of the system are shown in Figure 3. The applied pulse and the response of the system to the pulse for the axial component of the system are presented. The duration of the pulse was 600 *μ*s, which was of the order of the period of the aerodynamic impulse during an actual test in the tunnel. The system response function *g*(*t*) in time domain for the axial component is presented in Figure 4. The system response function has high frequency components that would occur during the practical implementation of the system in the shock tunnel and hence is a true representation of the system's practical impulse response. The presented *g*(*t*) is an average function representing a range of *a*(*t*) and *F*(*t*) that the model would be subjected to, during the current phase of shock tunnel operations.

**Figure 5:**
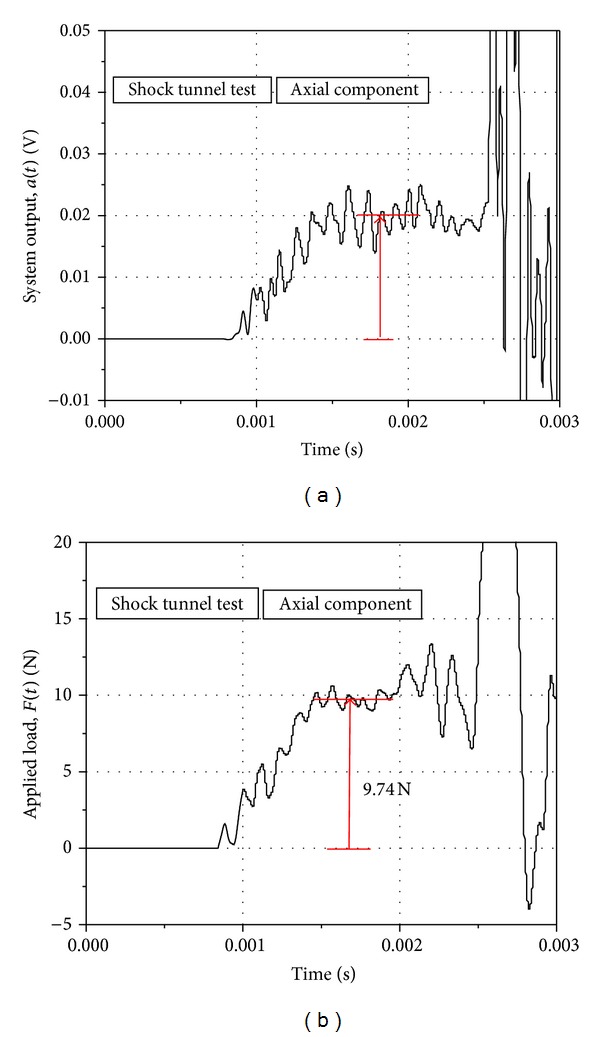
(a) System output and (b) deconvoluted applied load for axial component for aerodynamic test in shock tunnel. Model AOA = 10°.

**Figure 6:**
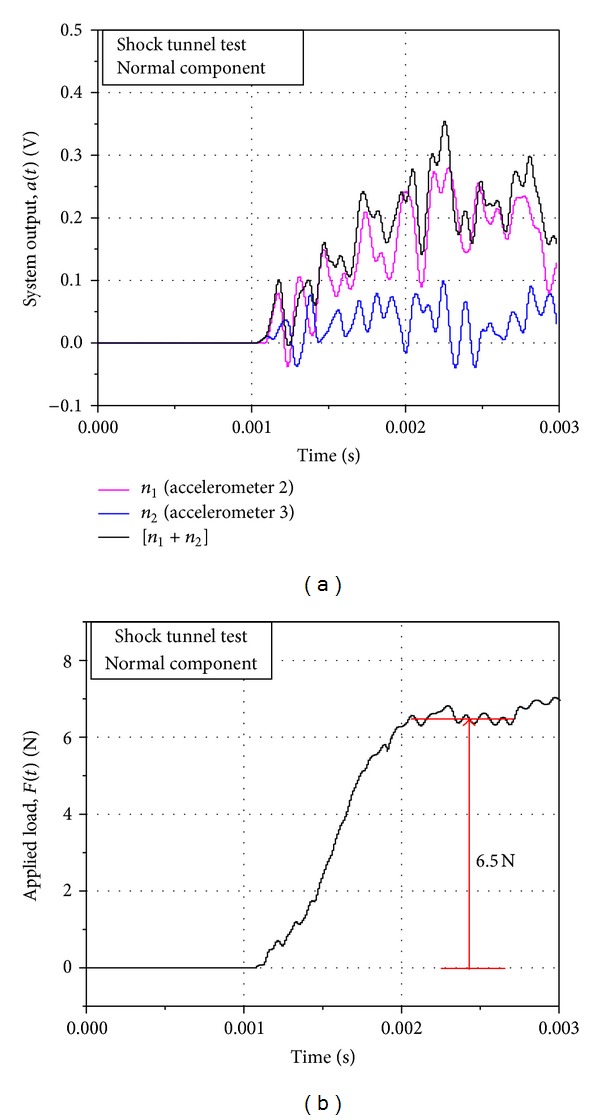
(a) System output and (b) deconvoluted applied load for normal component for aerodynamic test in shock tunnel. Model AOA = 10°.

**Figure 7:**
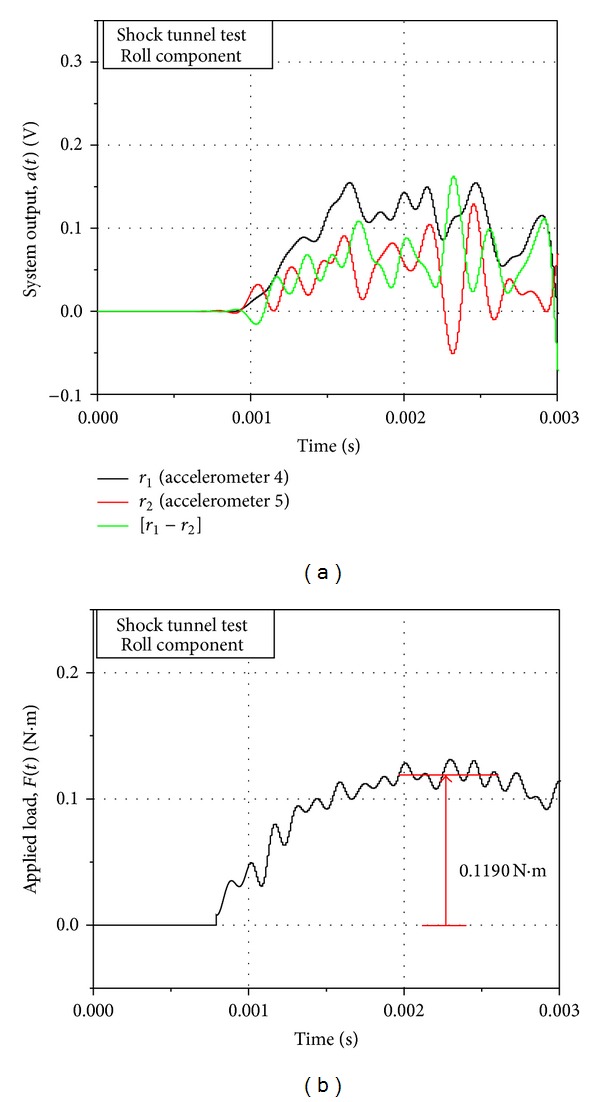
(a) System output and (b) deconvoluted applied load for rolling component for aerodynamic test in shock tunnel. Model AOA = 10°.

A practical, axial acceleration-time history (in terms of voltage) obtained from the system in the shock tunnel during an aerodynamic operation is presented in Figure 5(a). The deconvoluted axial force-time history corresponding to this acceleration signal is shown in Figure 5(b). Impulse loads were applied to the system to generate system response functions for normal force and rolling moment through the calibration procedure described above, and the practical system output from the tunnel was deconvoluted using the system response functions to obtain the applied loads. Figures 6 and 7 present the typical signals of the system output and the recovered applied load corresponding to normal force and rolling moment on the system, respectively, during an aerodynamic test in the tunnel. Two accelerometers each were used to capture the normal force and the rolling moment as discussed above, and hence the system output contains two signals in each case. The net system output for the force was obtained by matrix addition of the two, respective output signals, while the matrix subtraction of the two, respective output signals yielded the net system output for the moment. The deconvoluted signals exhibited sufficient, readable, steady magnitudes of the applied loads within the indicated test time of the tunnel.

**Figure 8:**
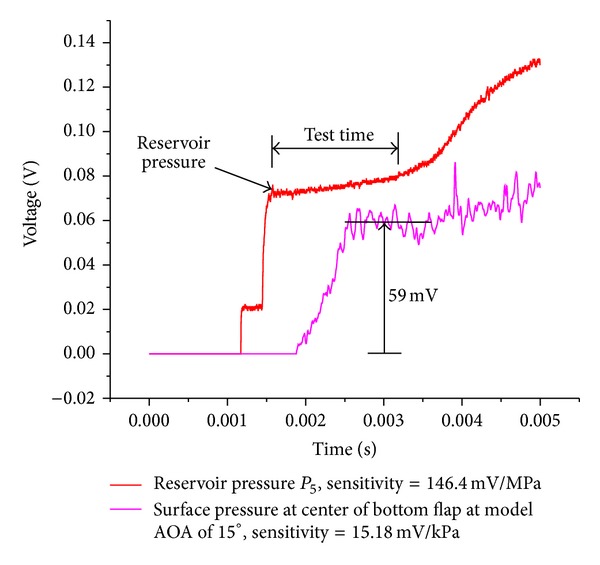
Typical pressure traces obtained during a test in shock tunnel.

**Figure 9:**
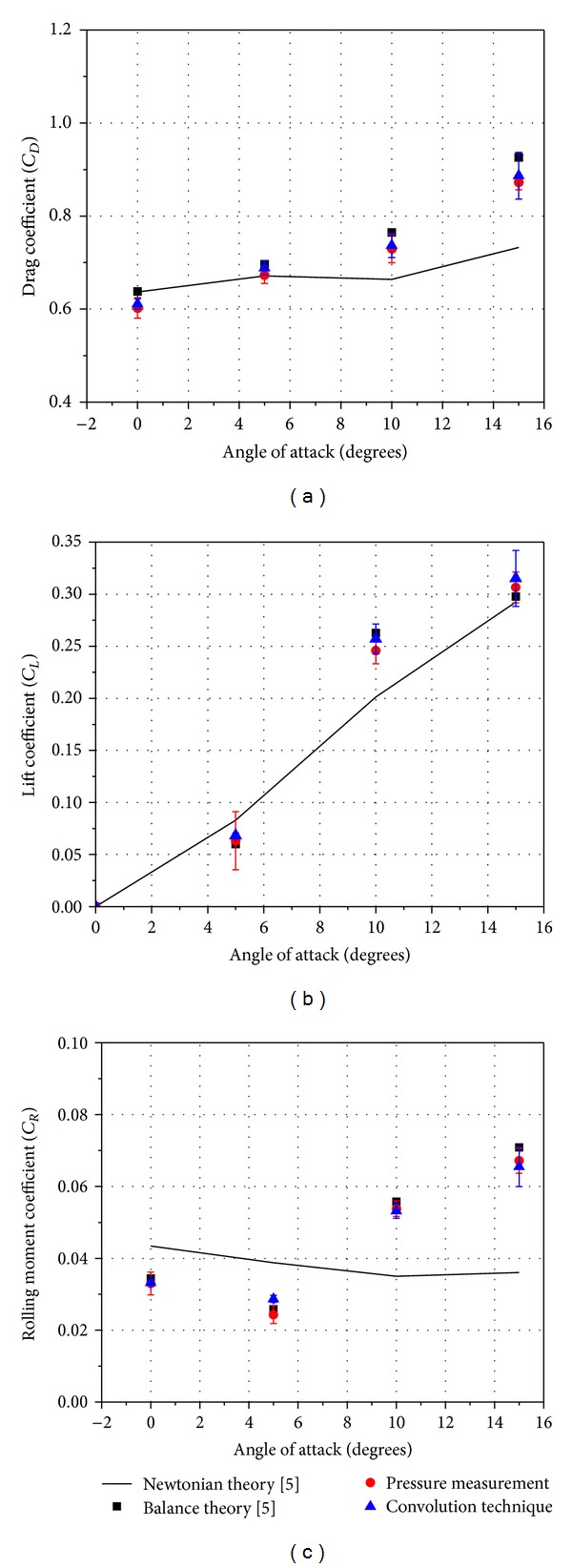
Variation of force and moment coefficients with model AOA. Scatter bars indicate standard deviation.

Figure 8 presents the representative signals from the pressure transducers in the shock tube reservoir and a location in the model surface, respectively. The reservoir pressure signal is included to indicate the typical, steady time available for the tests in the tunnel.  Figure 9 presents the variation of the coefficients of drag, lift, and rolling moment on the model with its angle of attack (AOA). The results are of two experimental procedures adopted in the present study and of an experimental and a theoretical procedure followed and reported previously [5]. The plots display a close agreement among the experimental results. The Newtonian theory is an inviscid, hypersonic flow theory, which could not account for certain extreme viscous effects in the flow field of the model, especially at higher AOA. Hence, the results of Newtonian theory differ slightly from the experimental results. The test model is a lifting, aerodynamic configuration, and the trends of the force and moment coefficients observed in Figure 9 are consistent with those of the blunt, lifting, hypersonic configurations under the given test conditions. The data presented was observed to be repeatable within the experimental scatter indicated on the data points.

The estimated uncertainties in the measured data are (Δ*C*_*D*_/*C*_*D*_) = ±4.57%, (Δ*C*_*L*_/*C*_*L*_) = ±4.04%, and (Δ*C*_*R*_/*C*_*R*_) = ±4.28%. The uncertainties are attributed to the error in the sensor sensitivities, derived freestream conditions and the results, output of the system, and the data acquisition system.

## 4. Concluding Remarks

An accelerometer-based force balance has been developed, calibrated, and used to measure drag, lift, and rolling moment on a blunt, flapped delta wing at a hypersonic Mach number in a shock tunnel. The magnitudes of the applied aerodynamic forces on the model have been derived using a convolution technique. The convolution technique used is independent of the sensor sensitivities, restraints of the system, and other interferences of the test facility during the operation. Hence, the data extracted through this method is believed to be accurate. The force and moment coefficients obtained through the method of convolution have been verified through a pressure measurement technique. A good agreement has been observed between the two sets of experimental data. The data generated agrees well with the data sets reported in the literature. The force balance developed is a useful tool in obtaining comprehensive data on complex geometries in ultrashort-duration, hypersonic test facilities.

## Figures and Tables

**Table 1:** Freestream conditions for tests in tunnel.

Test gas	Pressure (Pa)	Temperature (K)	Mach no.	Total enthalpy (MJ/kg)	Reynolds no. (/m)
Air	50.6	55.4	8	0.768	1.042 × 10^6^
(*γ* = 1.4)	±3.74%	±3.74%	±0.25%	±3.74%	±3.63%

**Table 2:** Properties of accelerometers.

Model (PCB)	Label in the test model (Figure 1)	Sensitivity (mV/(ms^−2^))	Operating frequency (kHz)	Peak load (g)
353B17	1	1.028	10	500
352C67	2	10.29	10	50
352C67	3	10.21	10	50
352C67	4	10.25	10	50
352C67	5	9.94	10	50
